# Current Trends in Mild Traumatic Brain Injury

**DOI:** 10.7759/cureus.18434

**Published:** 2021-10-02

**Authors:** Evan M Krueger, Anthony M DiGiorgio, Jonathan Jagid, Joacir G Cordeiro, Hamad Farhat

**Affiliations:** 1 Neurological Surgery, Carle Foundation Hospital, Urbana, USA; 2 Neurological Surgery, University of California San Francisco, San Francisco, USA; 3 Neurological Surgery, University of Miami, Coral Gables, USA; 4 Neurological Surgery, Advocate Aurora Health Care, Downers Grove, USA

**Keywords:** covid-19, prognosis, concussion, hemorrhage, mild traumatic brain injury

## Abstract

In this review, we provide an overview of the current research and treatment of all types of traumatic brain injury (TBI) before illustrating the need for improved care specific to mild TBI patients. Contemporary issues pertaining to acute care of mild TBI including prognostication, neurosurgical intervention, repeat radiographic imaging, reversal of antiplatelet and anticoagulation medications, and cost savings initiatives are reviewed. Lastly, the effect of COVID-19 on TBI is addressed.

## Introduction and background

Mild traumatic brain injury (TBI) is now commonly defined as a traumatic injury presenting a Glasgow coma scale (GCS) score of 13-15, although loss of consciousness and prior to admission amnesia are sometimes still described [1]. This term is often used interchangeably with 'concussion.' However, designating this as 'mild' may be a misnomer. As research on TBI continues to evolve, it is becoming clear that even mild TBI can have significant, life-altering consequences. A prospective analysis out of Transforming Research and Clinical Knowledge in TBI (TRACK-TBI) showed that 12 months after a mild TBI, 53% of patients reported significant functional limitations, defined as a Glasgow outcome scale extended (GOSE) scores <8 [2].

## Review

Current guidelines and research in all TBI

Professional organizations such as the American Association of Neurologic Surgeons and Congress of Neurologic Surgeons Section of Neurotrauma and Critical Care, National Health Institute funded TBI databases TRACK-TBI, and public outreach programs such as the ThinkFirst Foundation are at the forefront of improving outcomes for TBI patients. Their efforts have translated into decreased TBI mortality rates over the past decade in the United States [3]. Recently it was reported 75% of moderate TBI patients achieve GOSE scores ≥4 at 12 months, and 57% of severe TBI patients achieve GOSE scores ≥5 at six months [4, 5]. For clinicians, the Brain Trauma Foundation Guidelines for the Management of Severe TBI is an invaluable resource [6]. Recent updates to algorithms and approaches to the management of elevated intracranial pressure and decompressive hemicraniectomy have helped fill the gap between evidence and clinical practice [7, 8].

New areas of research will potentially change the management of TBI. These include biomarkers, treatment techniques, and medications. The 2018 A Prospective Clinical Evaluation of Biomarkers of Traumatic Brain Injury (ALERT-TBI) trial led to the first Food and Drug Administration (FDA) approved biomarkers for TBI such as ubiquitin C-terminal hydrolase-L1 (UCH-L1) and glial fibrillary acidic protein (GFAP) [9]. These results are promising and translational research is continuing to determine their optimal clinical usage [10, 11]. Abbott Diagnostic is now partnering with the United States Department of Defense and the TRACK-TBI consortium to conduct a multicenter, pivotal clinical trial on the i-STAT Point-of-Care version of UCH-L1/GFAP tandem plasma test for mild TBI [12]. Recognition and treatment of post-traumatic cerebral vasospasm appear to be important for secondary injury prevention [13, 14]. Middle meningeal artery embolization may represent a minimally invasive method to treat and prevent the recurrence of chronic subdural hematomas [14, 15]. The Clinical Randomisation of an Antifibrinolytic in Significant Head injury-3 (CRASH-3) trial examined prehospital tranexamic acid (TXA) for TBI, finding a risk reduction for mortality with TXA compared to placebo [16]. However, 35.8% of the study sample was from a single country, and head injury-related mortality was 6.6% for patients with GCS 9-15; thus the trial remains controversial [17]. Results from the Randomised Evaluation of Surgery with Craniectomy for patients Undergoing Evacuation of Acute Subdural Haematoma (RESCUE-ASDH) trial that examines clinical outcomes and cost-effectiveness between craniectomy versus craniotomy for acute subdural hematomas, and the results from the Brain Oxygen Optimization in Severe TBI, Phase 3 (BOOST3) trial that compares the safety and efficacy of treatment strategy guided by brain tissue oxygen monitoring and intracranial pressure measurements versus intracranial pressure measurements alone, are both eagerly awaited [18, 19].

Issues in mild TBI

Relatively few guidelines exist for hospital management of mild TBI despite that it accounts for 95% of all head injuries [20]. This has translated into variable acute care for mild TBI patients [21]. Furthermore, a large percentage of mild TBI patients do not receive adequate discharge instructions or follow-up [22]. Hence there is currently a tremendous opportunity and need for neurosurgeons to spearhead the improvement in the care of mild TBI patients.

Recent attention has been given to simple clinical measures with prognostic value to help guide clinical follow-up. For computed tomography (CT) positive mild TBI patients, intraventricular and/or petechial hemorrhages are particularly associated with worse long-term outcomes (GOSE <5, OR: 3.47), while epidural hematomas are not [23]. Pre-existing psychiatric conditions are risk factors for worse functional outcomes, increased incidence of post-injury posttraumatic stress disorder and major depressive disorder, and suicidal ideation [24-26]. Patients may need long-term, targeted, and pro-active rehabilitative services. It was recently shown that three-month return to work status and post-concussion symptoms are predictive of six-month return to work status [27].

The need for neurosurgical intervention in mild TBI patients is generally uncommon. In 2019, a large, single-center retrospective review reported that, in isolated CT positive mild TBI patients, overall neurosurgical intervention rates were 9.4% [28]. This was higher than the reported rate of 3.5% from a previous meta-analysis published in 2018 for non-isolated CT positive mild TBI patients [29]. Delayed deterioration necessitating neurosurgical intervention on initially non-surgical CT positive mild TBI patients is a feared complication that often influences decisions to escalate care; however, the incidence of this deterioration has been reported to range from 1.4-1.5% [28, 30]. Deterioration is reported to be more common in the elderly; potentially due to the larger subdural space and delayed hyperemia and hyperperfusion [31]. Prevalent usage of anticoagulant and antiplatelet medications in the elderly was commonly deemed as culprits for this deterioration, but current evidence suggests otherwise [32, 33]. Reasons for these conflicting data remain unclear.

Pooled data has reported that isolated traumatic subarachnoid hemorrhage to have an even lower, 0.0017% chance of neurosurgical intervention [34]. This has led some to propose the mere presence of intracranial hemorrhage, in itself, is not sufficient to warrant interhospital transfer [35]. Therefore telemedicine, allowing consulting neurosurgeons to discuss a patient and directly review images, remains an important and evolving aspect of mild TBI care.

Another controversial point that has received attention in recent literature is the role of repeat CT scans in CT positive mild TBI patients. Krueger et al. reported that in CT positive mild TBI patients, only 11.2% of patients had a worsened repeat CT and that the clinical neurologic exam was the more sensitive and specific way to monitor patients [28]. A 2013 meta-analysis concluded a repeat CT is unnecessary in mild TBI patients who are stable or clinically improving [36]. The appropriate duration of neurologic exam monitoring to prevent morbidity still remains uncertain. Nonetheless, a repeat CT may serve utility in terms of providing a clearly defined uniform hospital policy, medical-legal protection, justification for intrahospital transfer to lower financial cost units or discharge, or justification for resuming anticoagulation medication. The decision to repeat imaging in mild TBI patients remains at the discretion of the individual treating physicians.

The optimal management for CT positive TBI patients taking oral antiplatelet and anticoagulation medication is not clear. Conflicting evidence surrounds the utility of platelet transfusions for patients taking pre-injury antiplatelet medications [37, 38]. Vitamin K antagonists appear to be associated with worse outcomes compared to other direct oral anticoagulants, despite that vitamin K antagonists more commonly receive reversal agents [39]. If given appropriate reversal agents, the use of preoperative anticoagulant medication does not result in increased postoperative bleeding risk in emergent surgical TBI patients [40]. Proving clinical equipoise in high risk, emergency situations is difficult. To our knowledge, no randomized control trials have addressed antiplatelet or anticoagulants reversal agents in surgical or non-surgical CT positive mild TBI patients. Unless contrary robust data becomes available, judicious use of reversal agents will likely remain common practice.

Clearly identifying low-risk mild TBI patients early in triage may be an important branch point during decision making, and represent an opportunity to improve care and lower costs. A single-center, prospective study by Stippler et al. reported a safe, 71% reduction in CT imaging by allocating CT positive mild TBI patients who were not taking anticoagulants or antiplatelets and without active epidural or subdural hemorrhages >1 cm, to receive six hours of close neurologic observation only. There were no missed injuries or delayed surgery using this protocol [41]. Yun et al. designed a single-center, prospective study for low-risk CT positive mild TBI patients who were seen in the emergency department that showed safety, efficacy, and reduced admission rates [42]. Another prospective study showed no differences in six-month outcomes for mild TBI patients admitted to various levels of care [43]. In a controversial article, a prospective study proposed a protocol excluding neurosurgeons in the acute management of nonsurgical TBI as a way to reduce cost [44]. A statement from the American Association of Neurologic Surgeons and Congress of Neurologic Surgeons Section of Neurotrauma and Critical Care argued for maintaining neurosurgical involvement in TBI [45]. We strongly reaffirm their statement, recognize neurosurgeons' unique training in all nuances of TBI care, and support their role in caring for the entire spectrum of TBI patients.

COVID-19 and TBI

The COVID-19 pandemic has brought forward unique challenges in managing TBI [46]. The virus appears to affect the central nervous system, but much remains to be learned [47]. Fortunately, emergent operative cases are still being prioritized. However, neurosurgeons are having to adapt and develop novel case triage algorithms [48]. Neurosurgeons may also be forced to consider the appropriate hospital disposition of low-risk CT positive mild TBI patients in lieu of limited availability of intensive care unit beds. These issues have brought renewed focus to the neurosurgeon’s role in the neurointensive care unit. Some have proposed the creation of a new subspecialty, acute care neurosurgery, to better integrate critical care into modern neurosurgery and allow neurosurgeons to focus specifically on emergencies such as TBI [49].

## Conclusions

Care for mild TBI continues to evolve, and hinges on high-quality translational research. Challenges remain in improving outcomes, lowering costs, and improving efficiency. Future evidence-based care guidelines for mild TBI patients can help achieve these goals.
